# p53-associated regulation of COX17 enhances elesclomol-induced copper-associated cytotoxicity and suppresses gastric cancer growth

**DOI:** 10.3389/fimmu.2026.1817305

**Published:** 2026-06-15

**Authors:** Qianling Li, Xiaoyin Dong, Yu Zhao, Yun Shen, Chenyang Yang, Yu Xi

**Affiliations:** 1 The First Affiliated Hospital of Shihezi University, Shihezi, China; 2 Department of Gastrointestinal Surgery, The First Affiliated Hospital of Shihezi University, Shihezi, China; 3 Department of Gastroenterology, The First Affiliated Hospital of Shihezi University, Shihezi, China

**Keywords:** angiogenesis-related phenotype, copper metabolism, COX17, cuproptosis, elesclomol, gastric cancer, p53

## Abstract

**Background:**

Cuproptosis is a copper-dependent form of regulated cell death with emerging relevance to cancer therapy. However, the upstream determinants of copper-associated cytotoxicity and cuproptosis-related sensitivity in gastric cancer remain incompletely defined.

**Methods:**

Using AGS gastric cancer cells with genetic gain- and loss-of-function of COX17 and p53, we assessed viability using CCK-8 assays, cell death using TUNEL staining, intracellular copper levels, migration and invasion using wound healing and Transwell assays, and the expression of copper handling and cuproptosis-associated proteins by Western blotting. The potential transcriptional regulation of COX17 by p53 was evaluated using ChIP-qPCR. Paracrine effects on angiogenesis-related phenotypes were evaluated in HUVECs exposed to tumor-cell-conditioned media, followed by tube formation, ROS, GSH, SOD, and MDA measurements. *In vivo* relevance was examined in a subcutaneous AGS xenograft model treated with elesclomol.

**Results:**

COX17 alone had only a modest effect on basal cell viability but markedly increased the sensitivity of AGS cells to elesclomol-induced copper-associated cytotoxicity, accompanied by increased intracellular copper accumulation and TUNEL positivity, whereas COX17 knockdown attenuated these effects. Elesclomol treatment with COX17 modulation was associated with increased SLC31A1 expression and decreased ATP7A expression. p53 overexpression suppressed malignant phenotypes and increased COX17 expression. ChIP-qPCR showed the enrichment of p53 at the COX17 promoter region, suggesting that p53 may participate in the transcriptional regulation of COX17. Functional rescue experiments further indicated that COX17 is an important downstream mediator of p53-associated sensitivity to elesclomol. Conditioned media from elesclomol-treated, COX17-overexpressing tumor cells inhibited HUVEC tube formation and proliferation and increased oxidative stress. In xenografts, elesclomol reduced tumor growth, which was further suppressed by COX17 overexpression and partially attenuated by COX17 silencing, accompanied by increased tumor copper accumulation, upregulation of p53/COX17/SLC31A1, and downregulation of ATP7A.

**Conclusion:**

These findings suggest that the p53–COX17 axis links copper metabolism to elesclomol-associated antitumor activity in gastric cancer. COX17 may enhance copper accumulation, oxidative stress, tumor-cell-death-associated changes, and angiogenesis-related phenotypic inhibition. Further studies using canonical cuproptosis markers, copper chelator rescue experiments, additional gastric cancer cell lines with different p53 backgrounds, and clinical validation are required to confirm the translational significance of this pathway.

## 1 Introduction

Gastric cancer (GC) remains one of the leading causes of cancer-related death worldwide because of its high mortality rate, late-stage diagnosis, and limited response to conventional therapies in many patients (1–3). Although therapeutic strategies for gastric cancer have improved in recent years, chemotherapy and targeted therapies still show limited efficacy in a substantial proportion of patients with advanced disease (4, 5). Therefore, identifying novel molecular vulnerabilities and therapeutic strategies remains an important goal in gastric cancer research. In recent years, increasing attention has been paid to cellular metabolism and stress responses, particularly the roles of metal homeostasis and reactive oxygen species (ROS) in regulating cancer cell fate (6–8).

Copper, an essential trace element, plays a crucial role in cellular processes such as oxidative phosphorylation, angiogenesis, and redox homeostasis (9, 10). Dysregulation of copper metabolism has been associated with multiple pathological conditions, including cancer (11). Cuproptosis, a recently identified copper-dependent form of regulated cell death, is characterized by copper-induced mitochondrial stress and is closely linked to mitochondrial protein lipoylation and energy metabolism (12, 13). The discovery of cuproptosis has opened new possibilities for targeting cancer cells by exploiting their sensitivity to copper overload. However, the molecular determinants that regulate cellular sensitivity to copper-associated cytotoxicity and cuproptosis-related processes in gastric cancer remain insufficiently understood. The tumor suppressor protein p53 is a key regulator of cell fate in response to cellular stress. As a central hub controlling apoptosis, autophagy, cell cycle arrest, and DNA damage responses, p53 also modulates mitochondrial metabolism and redox homeostasis (14). However, the interplay between p53 signaling and copper metabolism in cancer remains poorly defined. Some studies suggest that p53 may influence mitochondrial function and metal-related metabolic pathways, raising the possibility that p53 could affect cellular responses to copper stress. COX17 is a copper chaperone responsible for copper delivery to mitochondrial cytochrome c oxidase and is essential for maintaining mitochondrial copper homeostasis (15, 16). COX17 is essential for maintaining mitochondrial copper homeostasis and, thus, cellular copper levels (17). Given the pivotal role of copper in mitochondrial metabolism and regulated cell death pathways, understanding the relationship between p53 and COX17 may provide insight into copper-associated vulnerabilities in gastric cancer.

In this study, we investigate the role of p53–COX17 signaling in regulating the response of gastric cancer cells to elesclomol-induced copper stress. We hypothesized that p53 may positively regulate COX17 expression, thereby enhancing copper accumulation and increasing the sensitivity of gastric cancer cells to elesclomol-induced copper-associated cytotoxicity. In addition, we examined whether COX17-associated changes in elesclomol-treated gastric cancer cells influence tumor growth and angiogenesis-related phenotypes using *in vitro* and *in vivo* models. By clarifying the potential involvement of the p53–COX17 axis in copper metabolism and elesclomol response, this study may provide a basis for further investigation of copper-targeted therapeutic strategies in gastric cancer.

## 2 Materials and methods

### 2.1 Cell lines and culture conditions

Human gastric adenocarcinoma AGS cells and human umbilical vein endothelial cells (HUVECs) were obtained from Xiamen Yimo Biological Technology Co., Ltd. (AGS: IM-H081; HUVEC: cat. no. IM-H205; Xiamen, China). AGS cells were cultured in AGS-specific complete medium (IM-H081-1, IMMOCELL, China) supplemented with 10% FBS (FSD500, Excell Bio, China) and 1% penicillin–streptomycin (P/S; 100×, C0222, Beyotime, China). The HUVECs were maintained in HUVEC-specific complete medium (IM-H205-1, IMMOCELL, China) containing 10% FBS and 1% P/S under the same conditions. All cells were grown in a humidified incubator at 37 °C with 5% CO_2_ and 95% relative humidity. The cells were passaged when they reached approximately 80% confluence using 0.25% trypsin (Gibco, China). Digestion was performed for approximately 1 min until the cells became round and detached, after which complete medium was added to terminate trypsinization, and the cells were reseeded for subsequent experiments.

### 2.2 Plasmids, siRNA, and transfection procedures

Plasmids for human COX17 overexpression (oe-COX17) and p53 overexpression (oe-p53), as well as the corresponding empty vectors, were constructed and provided by a commercial supplier (IMMOCELL, China). Small interfering RNAs targeting COX17 (si-COX17) and p53 (si-p53), together with their negative control siRNA (si-NC), were synthesized by a certified biotech company (China). All constructs and oligonucleotides were validated by sequencing before use.

For transient transfection, AGS cells were seeded in six-well plates and cultured until they reached 60%–70% confluence on the day of transfection. Plasmids/siRNAs were added into Opti-MEM (Gibco, USA) and mixed with Lipofectamine TM 3000 transfection reagent (Invitrogen, USA) following the manufacturer’s protocols: 2.0–2.5 µg for plasmid DNA or 50–100 nM for siRNA was used. After incubating for 10–15 min at room temperature, the transfection complexes were added dropwise to cells in fresh-serum-containing medium. The cells were then incubated under standard culture conditions (37 °C, 5% CO_2_) for 24–48 h before subsequent assays. Transfection efficiency and knockdown or overexpression of COX17 and p53 were confirmed by Western blotting. All experiments were performed using cells within a limited passage range to ensure comparability.

### 2.3 Drug treatment and experimental grouping

Elesclomol, a copper ionophore, was dissolved in DMSO as a stock solution and diluted in complete culture medium immediately before use. For all *in vitro* experiments, AGS cells were treated with elesclomol at a final concentration of 40 nM for 2 h. The drug-containing medium was replaced with fresh complete medium, and the cells were further incubated for an additional 24 h before subsequent analyses. The concentration and exposure time of elesclomol were selected based on preliminary dose– and time–response optimization experiments, in which this condition produced measurable cytotoxic effects while maintaining sufficient viable cells for downstream assays. For all *in vitro* assays, the final DMSO concentration did not exceed 0.1% (v/v), and an equal volume of DMSO was added to the control wells.

For COX17-related experiments in AGS cells, the following groups were established: vector control (vector), elesclomol alone (elesclomol), COX17 overexpression alone (oe-COX17), COX17 overexpression plus elesclomol (oe-COX17 + elesclomol), COX17 knockdown alone (si-COX17), and COX17 knockdown plus elesclomol (si-COX17 + elesclomol). These groupings were applied to assays of cell viability, intracellular copper content, cell death, and protein expression.

For the p53–COX17 axis experiments, AGS cells were divided into control (control), elesclomol alone, elesclomol with p53 overexpression (elesclomol + oe-p53), elesclomol with p53 knockdown (elesclomol + si-p53), elesclomol with p53 overexpression plus COX17 silencing (elesclomol + oe-p53 + si-COX17), and elesclomol with p53 knockdown plus COX17 overexpression (elesclomol + si-p53 + oe-COX17). These conditions were used to evaluate cell viability, copper accumulation, cell death, and cuproptosis-associated protein expression, thereby dissecting the functional interaction between p53 and COX17.

To assess the effect of tumor-cell-derived factors on angiogenesis-related phenotypes, conditioned media from AGS cells under different treatments were collected and applied to the HUVECs. The HUVECs were grouped as follows: control conditioned medium (control CM), conditioned medium from elesclomol-treated AGS cells (elesclomol CM), conditioned medium from oe-COX17 + elesclomol-treated AGS cells (oe-COX17 + elesclomol CM), and conditioned medium from si-COX17 + elesclomol-treated AGS cells (si-COX17 + elesclomol CM). Conditioned media were collected after AGS cells had undergone elesclomol treatment (40 nM for 2 h followed by 24 h of incubation in fresh medium). These groups were used in tube formation, CCK-8, ROS, and oxidative stress assays. Because the conditioned medium approach cannot fully exclude all indirect or residual-drug-related effects, the results were interpreted as angiogenesis-related phenotypic changes rather than definitive mechanistic proof of anti-angiogenesis.

For *in vivo* studies, tumor-bearing nude mice were randomly assigned to four groups: DMSO vehicle control (DMSO), elesclomol alone (elesclomol), COX17 overexpression plus elesclomol (oe-COX17 + elesclomol), and COX17 knockdown plus elesclomol (si-COX17 + elesclomol). Drug or vehicle was administered according to the predefined schedule, and body weight, tumor volume, histology, tissue copper content, cytokine levels, and protein expression were analyzed at the end of treatment.

### 2.4 Cell viability assay

Cell viability was measured using Cell Counting Kit-8 (CCK-8) according to the manufacturer’s instructions. Briefly, AGS cells were seeded into 96-well plates at a density of 3–5 × 10^3^ cells/well in 100 µL of complete medium and cultured overnight at 37 °C with 5% CO_2_. At 24 h after plasmid or siRNA transfection and/or elesclomol treatment as described above, 10 µL of CCK-8 solution was added to each well and gently mixed. The cells were then incubated at 37 °C for 1 to 2 h. Absorbance at 450 nm was measured using a microplate reader (Thermo Fisher Scientific, USA). Wells containing the medium and CCK-8 reagent without cells were used as blanks, and their absorbance values were subtracted from the sample values. Cell viability was expressed as a percentage of the respective control group. Each experimental condition was analyzed using at least three technical replicates, and experiments were repeated in at least three independent biological replicates.

### 2.5 Intracellular copper ion measurement

Intracellular copper ion concentration was determined using a colorimetric copper assay kit according to the manufacturer’s instructions. Briefly, 2 × 10^6^ AGS cells were seeded in six-well plates and treated with elesclomol alone, COX17 overexpression or knockdown, or COX17 overexpression or knockdown combined with elesclomol as described above. After treatment, the cells were washed twice with ice-cold PBS and collected using a cell scraper. The cell pellets were resuspended in lysis buffer (Beyotime, China) containing protease inhibitors, and total protein concentration was determined using BCA Protein Assay Kit (Thermo Fisher Scientific, USA). Copper ions were extracted using the copper-specific buffer provided by the assay kit and measured by colorimetric analysis. The assay was based on the formation of a stable complex between Cu^2+^ and a chromogenic reagent, with absorbance measured at 570 nm. A standard curve was generated using known copper concentrations, and the copper levels in the cell samples were calculated accordingly.

### 2.6 TUNEL staining for cell death

Cell death was evaluated using a terminal deoxynucleotidyl transferase dUTP nick end labeling (TUNEL) assay according to the manufacturer’s protocol. TUNEL staining was used to detect DNA fragmentation and was not interpreted as a specific marker of cuproptosis. AGS cells were seeded onto coverslips in 24-well plates at a density of 1 × 10^4^ cells/well. After incubation, the cells were treated with elesclomol alone, COX17 overexpression, COX17 knockdown, or COX17 overexpression/knockdown combined with elesclomol, as described above. After 24–48 h of treatment, the cells were washed twice with cold PBS and fixed with 4% paraformaldehyde for 15 min at room temperature. The cells were then permeabilized with 0.2% Triton X-100 (Beyotime, China) for 5 min. Subsequently, the cells were incubated with TUNEL reaction mixture containing terminal deoxynucleotidyl transferase (TdT) and fluorescein-dUTP in a humidified chamber at 37 °C for 1 h. The reaction was terminated using the stop buffer supplied with the kit. Nuclei were counterstained with DAPI at 0.5 μg/mL for 5 min. TUNEL-positive cells were quantified by counting at least 200 cells per well. Three independent biological replicates were performed for each treatment group.

### 2.7 Western blot analysis

The protein expression levels of COX17, p53, SLC31A1, ATP7A, and GAPDH were analyzed by Western blotting. AGS cells were seeded in six-well plates at approximately 2 × 10^6^ cells/well and treated with elesclomol, COX17 overexpression, COX17 knockdown, or the indicated combinations as described above. After treatment, the cells were washed twice with ice cold PBS. Total protein was extracted using RIPA lysis buffer (Biotean, China) supplemented with protease and phosphate inhibitors (Roche, Switzerland). Protein concentration was measured using BCA Protein Assay Kit (Thermo Fisher Scientific, USA). Equal amounts of protein were separated by SDS-PAGE using 10% or 12% gels, depending on the molecular weight of the target proteins, and then transferred onto PVDF membranes (Merck MilliPore, US) using a wet transfer system (BioRad, US) at 100 V for 1 h. After transfer, the membranes were blocked with 5% nonfat milk in TBST (Tris-buffered saline with 0.1% Tween-20) for 1 h to reduce nonspecific binding. The membranes were then incubated overnight at 4 °C with the following primary antibodies: anti-COX17 (1:1,000; Abcam, UK), anti-p53 (1:1,000; Santa Cruz Biotechnology, USA), anti-SLC31A1 (1:1,000; Thermo Fisher Scientific, USA), anti-ATP7A (1:1,000; Abcam, UK), and anti-GAPDH (1:5,000; Proteintech, China). After washing with TBST, the membranes were incubated with HRP-conjugated secondary antibodies (1:5,000; Jackson ImmunoResearch, USA) at room temperature. Protein bands were visualized using an ECL chemiluminescence detection kit (Thermo Fisher Scientific, USA) and detected with a chemiluminescence imaging system (Bio-Rad, USA). Densitometrical analyses were performed using ImageJ software (NIH, USA), and protein expression levels were normalized to GAPDH.

### 2.8 Wound healing and Transwell assays

Cell migration was observed using a wound healing assay. AGS cells were seeded into six-well plates at a density of 2 × 10^6^ cells/well and cultured overnight until they reached approximately 90% confluence. A straight scratch was created through the cell monolayer using a 200 μL pipette tip. After wounding, the cells were washed twice with PBS to remove detached cells and then treated with elesclomol, COX17 overexpression, COX17 knockdown, or the indicated combinations. The cells were cultured in serum-less medium to minimize the influence of cell proliferation on wound closure. Images of the wound area were captured using a light microscope (Olympus, Japan, 100 times) at ×100 magnification at 0 and 24 h. Migration was quantified by measuring the wound width at multiple points. The wound closure rate was calculated as follows: wound closing(%) = {(initial wound area - remaining wound area)/initial wound area} × 100%.

The invasive ability of AGS cells was evaluated using a Transwell assay. Briefly, a Transwell chamber (Corning, USA) with 8 μm pore size was pre-coated with 50 µL Matrigel (Corning, USA) at 37 °C for 30 min. After coating, 1 × 10^6^ AGS cells were resuspended in 200 µL serum-free medium and added to the upper chamber; while 600 µL complete medium containing 10%FBS was added to the lower chamber as a chemoattractant. The cells were cultured in a humidified incubator at 37 °C with 5% CO_2_ for 24–48 h. Non-invading cells on the upper surface of the membrane were gently removed with a cotton swab. Cells that had invaded the lower surface of the membrane were fixed with 4% paraformaldehyde for 10 min and stained with crystal violet at room temperature for 10 min. Images were captured using a light microscope at ×200 magnification. The invading cells were counted in five randomly selected fields per group, and the average value was used for analysis.

### 2.9 Chromatin immunoprecipitation–qPCR

Chromatin immunoprecipitation followed by qPCR (ChIP-qPCR) was performed to examine the potential binding of p53 to the COX17 promoter region. AGS cells were seeded in 10-cm dishes and transfected with oe-p53 or empty vector as described above. At 48 h after transfection, the cells were fixed with 1% formaldehyde at room temperature for 10 min to cross-link protein–DNA complexes. The reaction was stopped by adding 0.125 M glycine, and the cells were washed with ice-cold PBS. Cells were collected in ChIP lysis buffer (Beyotime, China), and chromatin was sheared into fragments of approximately 200–500 bp by sonication using a Bioruptor system (Diagenode, Belgium) at 4 °C for 10 min with 30 s on/off cycles. The sheared chromatin was precleared with protein A agarose beads (Invitrogen, USA) for 1 h at 4 °C to reduce nonspecific binding. The chromatin samples were then incubated overnight at 4 °C with anti-p53 antibody (Santa Cruz Biotechnology, USA) or control IgG (Cell Signaling Technology, USA). Immune complexes were captured with protein A agarose beads, washed repeatedly with ChIP wash buffer, and eluted using ChIP elution buffer. Cross-links were reversed by incubating the samples at 65 °C for 4 h, followed by DNA purification using a PCR purification kit (Qiagen, Germany). For the qPCR analysis, primers were designed to amplify the COX17 promoter region containing predicted p53-binding sites. The primer sequences were as follows: COX17 promoter forward: 5′- GAGGCTCAGGAGAAGAAGCC-3′; COX17 promoter reverse: 5′- TTGTGGGCCTCGATGAGATG-3′. qPCR was performed using SYBR Green PCR Master Mix (Thermo Fisher Scientific, USA) on a QuantStudio 6 Real-Time PCR System (Thermo Fisher Scientific, USA). COX17 promoter fragment enrichment was normalized to input DNA and calculated using the 2^ΔΔCt^ method. Importantly, ChIP-qPCR was used to assess p53 enrichment at the COX17 promoter region. The assay was not intended to demonstrate DNA binding by COX17 itself, as COX17 is not a conventional transcription factor.

### 2.10 Reactive oxygen species detection

Reactive oxygen species (ROS) levels in cells were detected using the fluorescent probe 2′,7′-dichlorodihydrofluorescein diacetate (DCFH-DA; Beyotime, China) and quantified based on fluorescence intensity. AGS cells were seeded into six-well plates at a density of 2 **×** 10^6^ cells/well and treated with elesclomol, COX17 overexpression or knockdown, or elesclomol combined with COX17 overexpression or knockdown as described above. After 24–48 h of treatment, the cells were washed twice with PBS and incubated with 10 µM DCFH-DA at 37 °C in the dark for 30 min. Intracellular esterases convert DCFH-DA to DCFH, which is then oxidized by ROS to fluorescent DCF. After incubation, the cells were washed twice with PBS to remove excess probe and resuspended in PBS. Fluorescence was measured using a flow cytometer (BD FACSCanto II, USA) with an excitation wavelength of 488 nm and an emission wavelength of 525 nm. For fluorescence microscopy, the cells were seeded on coverslips and treated as described above. After DCFH-DA staining, the cells were washed, mounted, and observed under a fluorescence microscope (Olympus, Japan) at ×400 magnification. Fluorescence intensity was quantified as mean fluorescence intensity using ImageJ software (NIH, USA).

### 2.11 Oxidative stress marker assays

To assess oxidative stress, glutathione (GSH), superoxide dismutase (SOD) activity, and malondialdehyde (MDA), a marker of lipid peroxidation, were measured using commercially available assay kits (Beyotime, China) according to the manufacturer’s instructions. AGS cells were seeded in six-well plates and treated as described above. After treatment, the cells were collected and lysed using the lysis buffers supplied with the kits. Total protein concentration was determined using BCA Protein Assay Kit (Thermo Fisher Scientific, USA). GSH and SOD assays were performed according to the manufacturer’s protocols, and absorbance was measured at 412 nm for GSH and 450 nm for SOD using a microplate reader (Thermo Fisher Scientific, USA). MDA levels were measured using the thiobarbituric acid reactive substances (TBARS) method. In this assay, MDA reacts with thiobarbituric acid to generate a pink chromophore, which was measured at 532 nm. MDA concentrations were calculated using a standard curve generated from known MDA concentrations.

### 2.12 Tube formation assay

Tube formation assays were performed to assess the angiogenesis-related capacity of HUVECs exposed to conditioned media from differently treated AGS cells. Briefly, 96-well plates were coated with 50 µL Matrigel and incubated at 37 °C for 30 min to allow gel polymerization. HUVECs were then seeded onto the Matrigel-coated wells, and 100 µL of conditioned medium was added to each well. The conditioned media included control CM, elesclomol CM, oe-COX17 + elesclomol CM, and si-COX17 + elesclomol CM. The cells were incubated at 37 °C with 5% CO_2_ for 6–8 h to allow tube-like structures to form. Images of the HUVEC network were captured under a light microscope (Olympus, Japan) at ×100 magnification. The number of branch points, total tube length, and number of closed loops were quantified using ImageJ software (NIH, USA). These assays were used to evaluate angiogenesis-related phenotypic changes *in vitro*.

### 2.13 Xenograft tumor model in nude mice

All animal experiments were approved by the Institutional Ethics Committee of The First Affiliated Hospital of Shihezi University and were performed in accordance with the Guide for the Care and Use of Laboratory Animals. Male BALB/c nu/nu mice, approximately 6–8 weeks old and weighing 18–22 g, were purchased from Beijing Vital River Laboratory Animal Technology Co., Ltd., (Beijing, China). The mice were maintained under standard conditions with 12-h light/dark cycle, ambient temperature of 22 °C–25 °C, and relative humidity of 40%–60%, with free access to food and water.

For the tumorigenesis assay, AGS cells (1 × 10^7^ cells in 100 µL PBS) transfected with oe-COX17, si-COX17, or the respective control vectors/si-NC were subcutaneously injected into the dorsal flank of nude mice. After tumors were established, the mice were randomly assigned to four groups: (1) DMSO (control group, *n* = 6), (2) elesclomol alone, *n* = 6, (3) COX17 overexpression + elesclomol (oe-COX17 + elesclomol, *n* = 6), and (4) COX17 knockdown + elesclomol (si-COX17 + elesclomol, *n* = 6). Elesclomol (10 mg/kg) was administered intraperitoneally every 3 days, starting when the tumors reached approximately 100 mm³, for a total treatment period of 21 days. The control mice received an equal volume of DMSO. Tumor volume was measured weekly using calipers and calculated according to the following formula: tumor volume = length × width^2/^2, where length refers to the largest diameter and width refers to the smallest diameter of the tumor.

At the end of treatment, the mice were euthanized, and tumors were excised, weighed, and processed for further analyses. Body weight was measured weekly to monitor systemic condition and potential treatment-related toxicity. The excised tumors were fixed with 4% paraformaldehyde overnight for histological examination, embedded in paraffin, and sectioned at 5 μm. The sections were stained with H&E to evaluate tumor morphology and necrosis. The copper content of tumor tissues was measured using a colorimetric assay kit, and serum inflammatory cytokine levels, including TNF-α, IL-6, IL-1β, and IL-18, were determined using enzyme-linked immunosorbent assays (ELISA). Protein expression in tumor lysates was analyzed by Western blotting as described above.

### 2.14 Histological analysis

Tumor morphology and necrosis in xenograft tumors were evaluated by histologic examination. At the end of treatment, excised tumor tissues were gently washed with cold PBS and fixed in 4% paraformaldehyde (Beyotime, China) for 24 h at room temperature. The tissues were dehydrated through a graded ethanol series and embedded in paraffin (Sigma-Aldrich, USA). Tissue sections were cut from paraffin-embedded samples using a Leica microtome (Germany) at a thickness of 5 µm. Tumor sections were deparaffined with xylene (Beyotime, China), rehydrated through decreasing concentrations of ethanol, and stained with hematoxylin and eosin (H&E; Beyotime, China). The sections were stained with hematoxylin for 5–10 min, washed in running tap water, and then stained with eosin for 2 to 3 min. Slides were dehydrated through increasing concentrations of ethanol, cleared with xylene, mounted with mounting medium (Beyotime, China), and covered with coverslips. Histological observations were performed using a light microscope (Olympus, Japan) at ×200 and ×400 magnification, respectively. Tumor morphology, necrotic areas, and structural damage were evaluated using ImageJ software (NIH, USA).

### 2.15 ELISA for cytokines

Serum levels of TNF-α, IL-6, IL-1β, and IL-18 were measured using commercial ELISA kits (BioLegend, USA) according to the manufacturer’s protocol. After the *in vivo* experiment, blood samples were collected by cardiac puncture under anesthesia, and serum was separated by centrifugation at 3,000 × *g* for 10 min at 4 °C. The serum samples were stored at -80 °C until analysis. For each cytokine, the serum samples were diluted according to the kit instructions and added in duplicate to 96-well plates pre-coated with the corresponding capture antibody. After incubation for 2 h at room temperature, the wells were washed four times with wash buffer. The corresponding biotin-labeled detection antibody was then added and incubated for 1 h. After washing, streptavidin–HRP was added to each well and incubated for 1 h. After washing, streptavidin–HRP was added to each well and incubated for 30 min at room temperature. Following the additional washing, TMB substrate solution was added and incubated for 15 min, after which the reaction was stopped using a stop solution. Absorbance was measured at 450 nm using a microplate reader (Thermo Fisher Scientific, USA). Cytokine concentrations were calculated using standard curves generated from recombinant cytokine standard.

### 2.16 Statistical analysis

Statistical analyses were performed using GraphPad prism 8.0 software (GraphPad Software, USA). Data were presented as means ± SD from at least three independent biological replicates, each with three technical replicates unless otherwise stated. Comparisons between two groups were performed using Student’s *t*-test. Comparisons among more than two groups were performed using a one-way ANOVA followed by Tukey’s *post hoc* test. A value of *P <*0.05 was considered statistically significant.

## 3 Results

### 3.1 COX17 regulates the sensitivity of gastric cancer cells to elesclomol-induced copper-associated cytotoxicity

**Figure 1:**
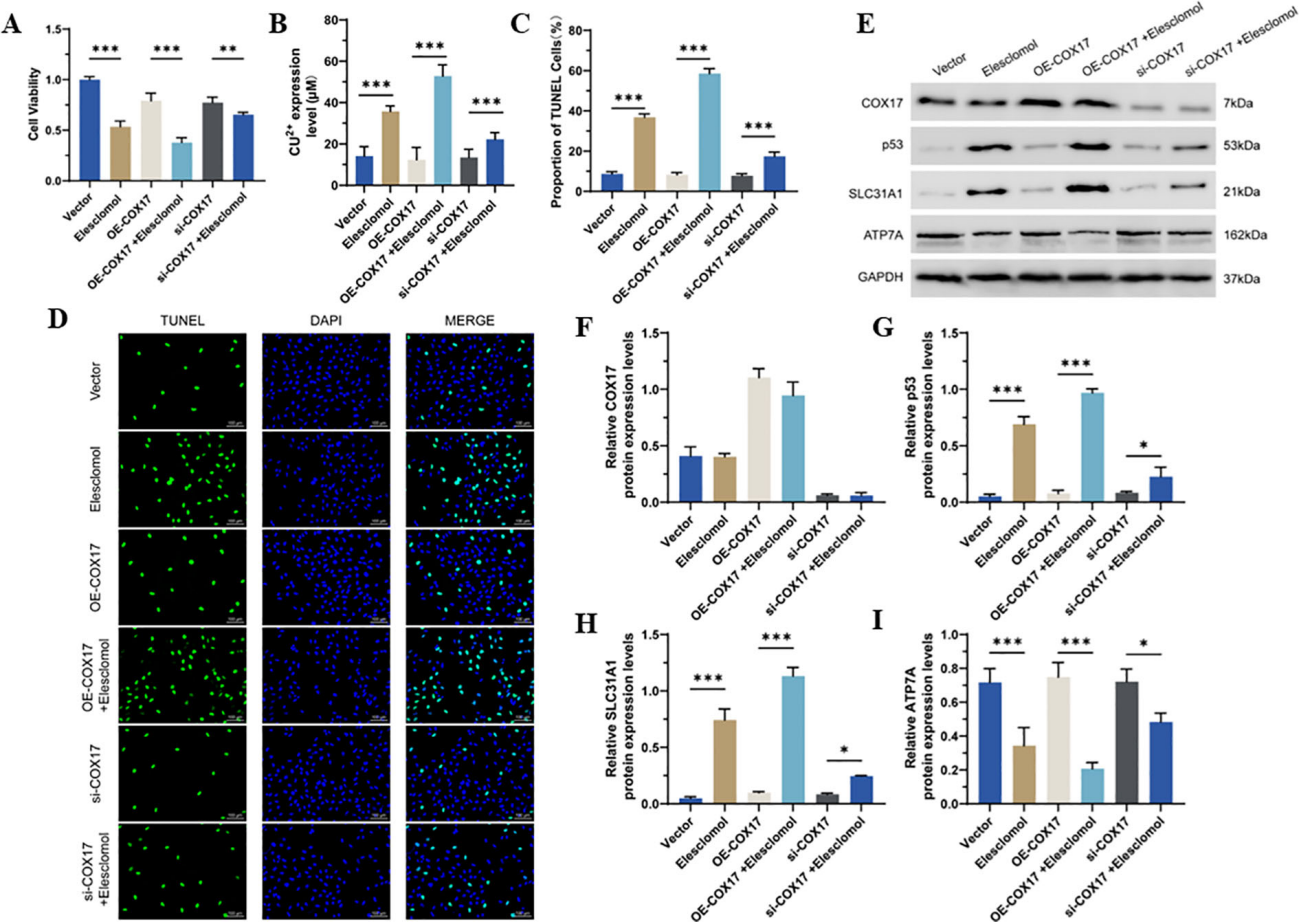
COX17 regulates the sensitivity of AGS gastric cancer cells to elesclomol-induced copper-associated cytotoxicity. **(A)** Cell viability of AGS cells measured by CCK-8 assay in the vector, elesclomol, COX17-overexpression (OE-COX17), OE-COX17 + elesclomol, COX17-knockdown (si-COX17), and si-COX17 + elesclomol groups. **(B)** Intracellular copper ion content detected by colorimetric assay in the indicated groups. **(C)** Representative TUNEL and DAPI fluorescence images showing apoptotic cells under the different treatments. Scale bar = 100 μm. **(D)** Quantification of the percentage of TUNEL-positive cells corresponding to **(C)**. **(E)** Representative Western blot images of COX17, p53, SLC31A1, ATP7A, and GAPDH in AGS cells from each treatment group. **(F–I)** Densitometric analysis of COX17 **(F)**, p53 **(G)**, SLC31A1 **(H)**, and ATP7A **(I)** protein levels normalized to GAPDH. Data are presented as mean ± SD (*n* = 3). Statistical significance is indicated as *P < 0.05, **P < 0.01, ***P < 0.001.

To investigate whether COX17 affects the response of gastric cancer cells to copper-associated cytotoxic stress, AGS cells were subjected to COX17 overexpression or knockdown and then treated with the copper ionophore elesclomol. The CCK-8 assay showed that elesclomol alone significantly decreased cell viability compared with the vector control group (*P* < 0.001). COX17 overexpression or knockdown alone caused only a modest reduction in cell viability (*P* < 0.001). In contrast, COX17 overexpression combined with elesclomol induced the most pronounced decrease in cell viability compared with elesclomol alone or COX17 overexpression alone (*P* < 0.001). COX17 knockdown partially attenuated the decrease in cell viability induced by elesclomol compared with elesclomol alone (**Figure 1A**).

Intracellular copper levels were next evaluated by colorimetric assay. Elesclomol treatment significantly increased the intracellular copper levels compared with the vector group (*P* < 0.001). In the presence of elesclomol, COX17 overexpression resulted in the highest intracellular copper accumulation among all groups (*P* < 0.001 versus OE-COX17 alone), whereas COX17 knockdown combined with elesclomol resulted in an intermediate increase that remained higher than that in the vector group. COX17 overexpression or knockdown alone did not markedly alter the copper content relative to the vector control (**Figure 1B**).

TUNEL staining was performed to evaluate cell death rather than to specifically identify cuproptosis. Few TUNEL-positive cells were observed in the vector, COX17 overexpression, and COX17 knockdown groups, with no obvious statistical difference among these groups. Elesclomol treatment significantly increased the number of TUNEL-positive cells compared with the vector group (*P* < 0.001). The combination of COX17 overexpression and elesclomol further increased the percentage of TUNEL-positive cells compared with COX17 overexpression alone (*P* < 0.001). However, COX17 knockdown plus elesclomol resulted in fewer TUNEL-positive cells than COX17-overexpression plus elesclomol, although the level remained significantly higher than in the vector group (*P* < 0.001) (**Figures 1C, D**).

Western blotting was performed to assess COX17 and copper-transport-related proteins. COX17 protein expression was increased in the COX17 overexpression and COX17 overexpression plus elesclomol groups compared with the vector group and was reduced in the COX17 knockdown and COX17 knockdown plus elesclomol groups (**Figures 1E, F**). Elesclomol treatment, particularly in combination with COX17 overexpression, was associated with significantly higher p53 and SLC31A1 levels, possibly reflecting enhanced copper-associated cellular stress responses (*P* < 0.001), whereas COX17 knockdown reduced their expression compared with the vector or elesclomol alone groups (**Figures 1E, G, H**). In contrast, ATP7A expression was relatively higher in the vector, COX17-overexpression, and COX17 knockdown groups and was significantly reduced after elesclomol treatment (*P* < 0.001), with the lowest levels observed in the COX17 overexpression plus elesclomol group (**Figures 1E, I**). These findings together suggest that COX17 enhances elesclomol-induced copper accumulation and cell-death-associated changes in AGS cells.

### 3.2 p53 positively regulates COX17 expression in gastric cancer cells

**Figure 2:**
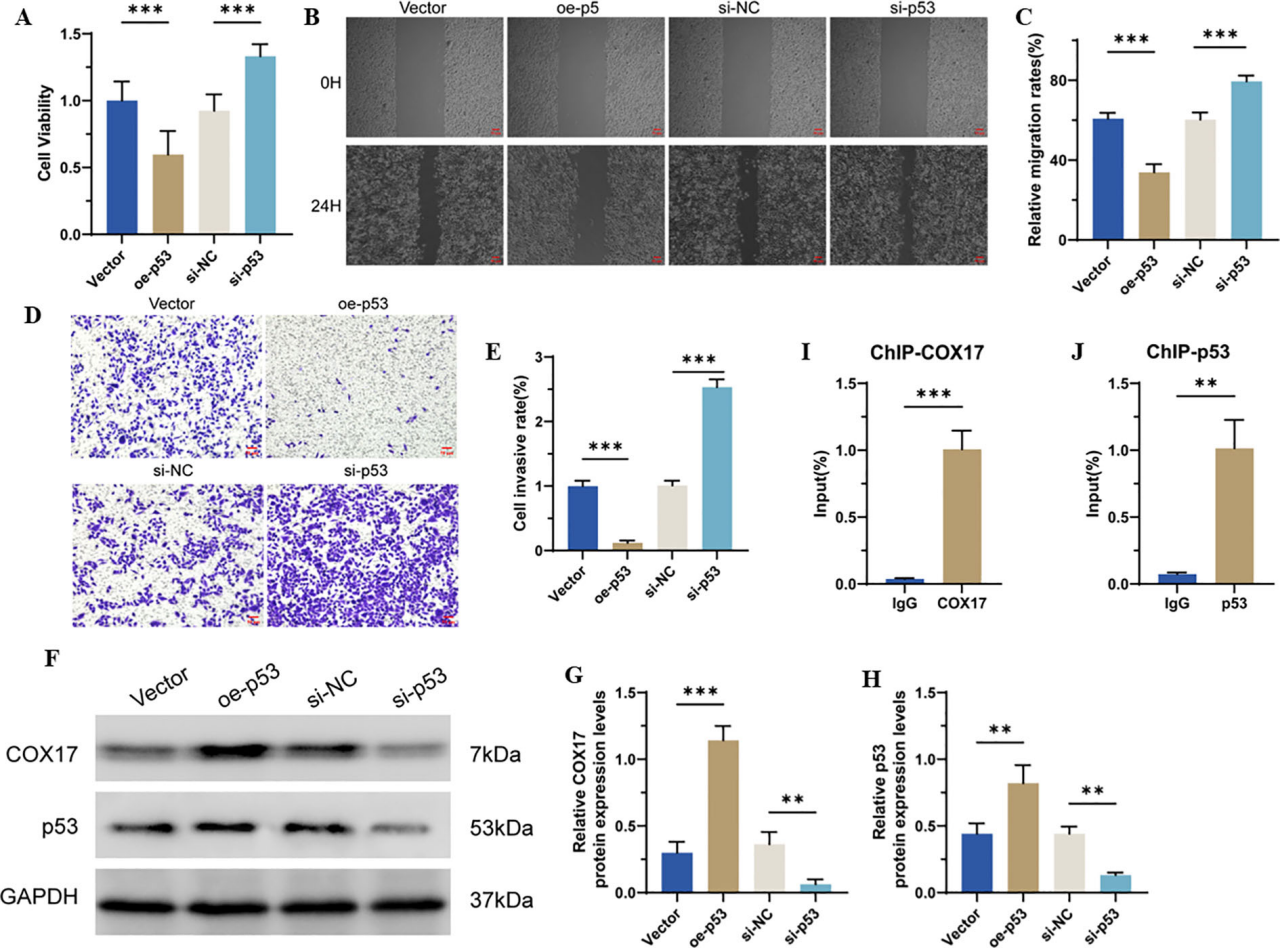
p53 positively regulates COX17 expression and suppresses the malignant phenotypes of gastric cancer cells. **(A)** Cell viability of AGS cells measured by CCK-8 after transfection with vector, oe-p53, si-NC, or si-p53 plasmids. **(B)** Representative images of wound-healing assays at 0 and 24 h. **(C)** Quantification of relative migration rates. **(D)** Representative images of Transwell invasion assays. **(E)** Quantification of invasion rates. **(F)** Western blot analysis of COX17 and p53 protein expression with GAPDH as loading control. **(G, H)** Densitometric quantification of COX17 and p53 protein levels. **(I, J)** ChIP-qPCR analysis showing the enrichment of COX17 promoter fragments precipitated by anti-p53 antibody compared with IgG control. Data are presented as mean ± SD (*n* = 3); **P < 0.01, ***P < 0.001.

To determine whether p53 regulates COX17 expression in gastric cancer, AGS cells were transfected with oe-p53 or si-p53 to overexpress or knock down p53. The CCK-8 assay showed that p53 overexpression significantly reduced the viability of AGS cells compared with the vector group (*P* < 0.001). Conversely, p53 silencing significantly increased cell viability compared with the si-NC group (*P* < 0.001) (**Figure 2A**).

Consistent with these findings, wound healing assays showed that oe-p53 cells exhibited slower migration at 24 h compared with vector controls cells (*P* < 0.001) (**Figures 2B, C**). Similarly, Transwell assays demonstrated that p53 overexpression markedly suppressed the invasive capacity of AGS cells (*P* < 0.001), whereas p53 knockdown significantly enhanced cell invasion compared with the corresponding control group (*P* < 0.001) (**Figures 2D, E**). In agreement with these phenotypic observations, Western blotting showed that p53 overexpression increased both p53 and COX17 protein levels compared with the vector control group (*P* < 0.001 and *P* < 0.01, respectively). Conversely, si-p53 transfection significantly decreased p53 and COX17 expression compared with si-NC (both *P* < 0.01) (**Figures 2F–H**). To further examine whether p53 is enriched at the COX17 promoter region, ChIP-qPCR was performed. The ChIP-qPCR analysis revealed significant enrichment of COX17 promoter fragments precipitated by anti-p53 antibody compared with IgG control (*P* < 0.001) (**Figures 2I, J**). These results suggest that p53 is enriched at the COX17 promoter region and may participate in the transcriptional regulation of COX17 expression in AGS cells. Collectively, these data suggest that p53 is associated with increased COX17 expression and may participate in its transcriptional regulation in AGS cells.

### 3.3 The p53–COX17 axis modulates elesclomol-induced copper-associated cytotoxicity and cuproptosis-related protein changes in gastric cancer cells

**Figure 3:**
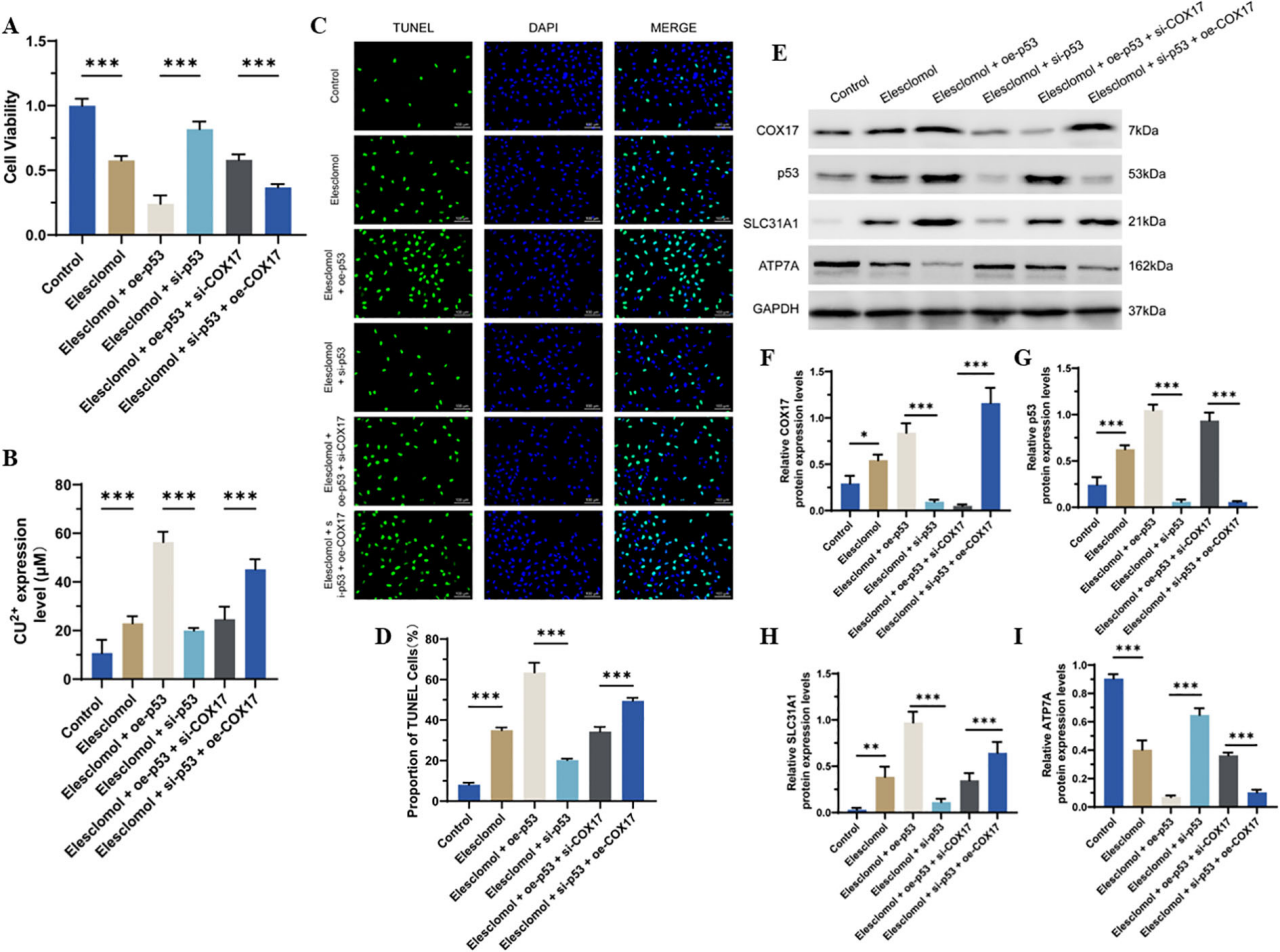
The p53–COX17 axis modulates elesclomol-induced copper-associated cytotoxicity and cuproptosis-associated protein changes in AGS gastric cancer cells. **(A)** CCK-8 assay showing cell viability in control, elesclomol, elesclomol + oe-p53, elesclomol + si-p53, elesclomol + oe-p53 + si-COX17, and elesclomol + si-p53 + oe-COX17 groups. **(B)** Intracellular Cu^2+^ levels measured by colorimetric assay in the indicated groups. **(C)** Representative TUNEL (green) and DAPI (blue) fluorescence images. Scale bar = 100 μm. **(D)** Quantification of TUNEL-positive cells. **(E)** Representative Western blots of COX17, p53, SLC31A1, ATP7A, and GAPDH. **(F–I)** Densitometric analysis of COX17 **(F)**, p53 **(G)**, SLC31A1 **(H)**, and ATP7A **(I)** normalized to GAPDH. Data are presented as mean ± SD (*n* = 3); *P < 0.05, **P < 0.01, ***P < 0.001.

To investigate whether COX17 contributes to the effect of p53 on elesclomol-induced cytotoxicity, AGS cells were first subjected to p53 overexpression or knockdown and then treated with elesclomol, with or without additional modulation of COX17 expression. The CCK-8 assay showed that elesclomol alone significantly decreased cell viability compared with the control group (*P* < 0.001). p53 overexpression further enhanced the cytotoxic effect of elesclomol, resulting in the lowest viability among all groups (*P* < 0.001). In contrast, p53 knockdown partially rescued cells from elesclomol-induced growth inhibition. Importantly, silencing COX17 in the context of p53 overexpression (elesclomol + oe-p53 + si-COX17) markedly attenuated the sensitizing effect of p53. Conversely, COX17 overexpression in p53-knockdown cells (elesclomol + si-p53 + oe-COX17) partially restored elesclomol sensitivity, leading to lower cell viability than that observed in the elesclomol + si-p53 group (*P* < 0.001) (**Figure 3A**).

Consistent with the differences in cell viability, the colorimetric analysis showed that elesclomol increased the intracellular Cu^2+^ levels compared with the control (*P* < 0.001), and this effect was further potentiated by p53 overexpression, resulting in the highest copper accumulation (*P* < 0.001). p53 knockdown markedly reduced elesclomol-induced copper accumulation (*P* < 0.001 vs. elesclomol + oe-p53). Conversely, COX17 overexpression in the p53-deficient background significantly increased the copper content compared with elesclomol + si-p53 (*P* < 0.001), suggesting that COX17 can partially compensate for the loss of p53 in promoting copper accumulation (**Figure 3B**).

TUNEL staining was used to evaluate cell death, not as a specific readout of cuproptosis. Very few TUNEL-positive cells were detected in the control group, whereas elesclomol alone markedly increased the number of TUNEL-positive cells compared with the control group (*P* < 0.001). This cell death-associated effect was further enhanced when elesclomol was combined with p53 overexpression, resulting in the highest TUNEL-positive rate among all groups (*P* < 0.001 vs. elesclomol). p53 knockdown substantially reduced elesclomol-induced TUNEL positivity (*P* < 0.001 vs. elesclomol + oe-p53). Consistent with the cell viability and copper accumulation data, COX17 knockdown largely blunted the pro-death effect associated with p53 overexpression (*P* < 0.01 vs. elesclomol + oe-p53), whereas COX17 upregulation in p53-silenced cells increased the number of TUNEL-positive cells compared with the elesclomol + si-p53 group (**Figures 3C, D**).

Western blot analysis further supported a functional p53–COX17 axis in regulating copper-transport- and cuproptosis-associated protein changes. Elesclomol treatment increased p53, COX17, and SLC31A1 expressions but decreased ATP7A expression compared with the control group (all *P* < 0.05). p53 overexpression further elevated p53 and COX17 expressions and was accompanied by significant induction of SLC31A1 and further reduction of ATP7A (all *P* < 0.001 vs. elesclomol alone). In contrast, p53 knockdown resulted in markedly lower levels of p53, COX17, and SLC31A1 and higher ATP7A expression (*P* < 0.001 vs. elesclomol + oe-p53). Notably, COX17 silencing in the p53-overexpression background reduced COX17 and SLC31A1 expression and partially reversed ATP7A downregulation, whereas COX17 overexpression in p53-deficient cells restored COX17 and SLC31A1 levels and again reduced ATP7A (**Figures 3E–I**). These findings together indicate that p53 may enhance elesclomol-induced copper-associated cytotoxicity partly through the upregulation of COX17 and that COX17 acts as an important downstream mediator of this response in AGS gastric cancer cells.

### 3.4 Conditioned media from COX17-mediated AGS cells affect angiogenesis-related phenotypes in HUVECs

**Figure 4:**
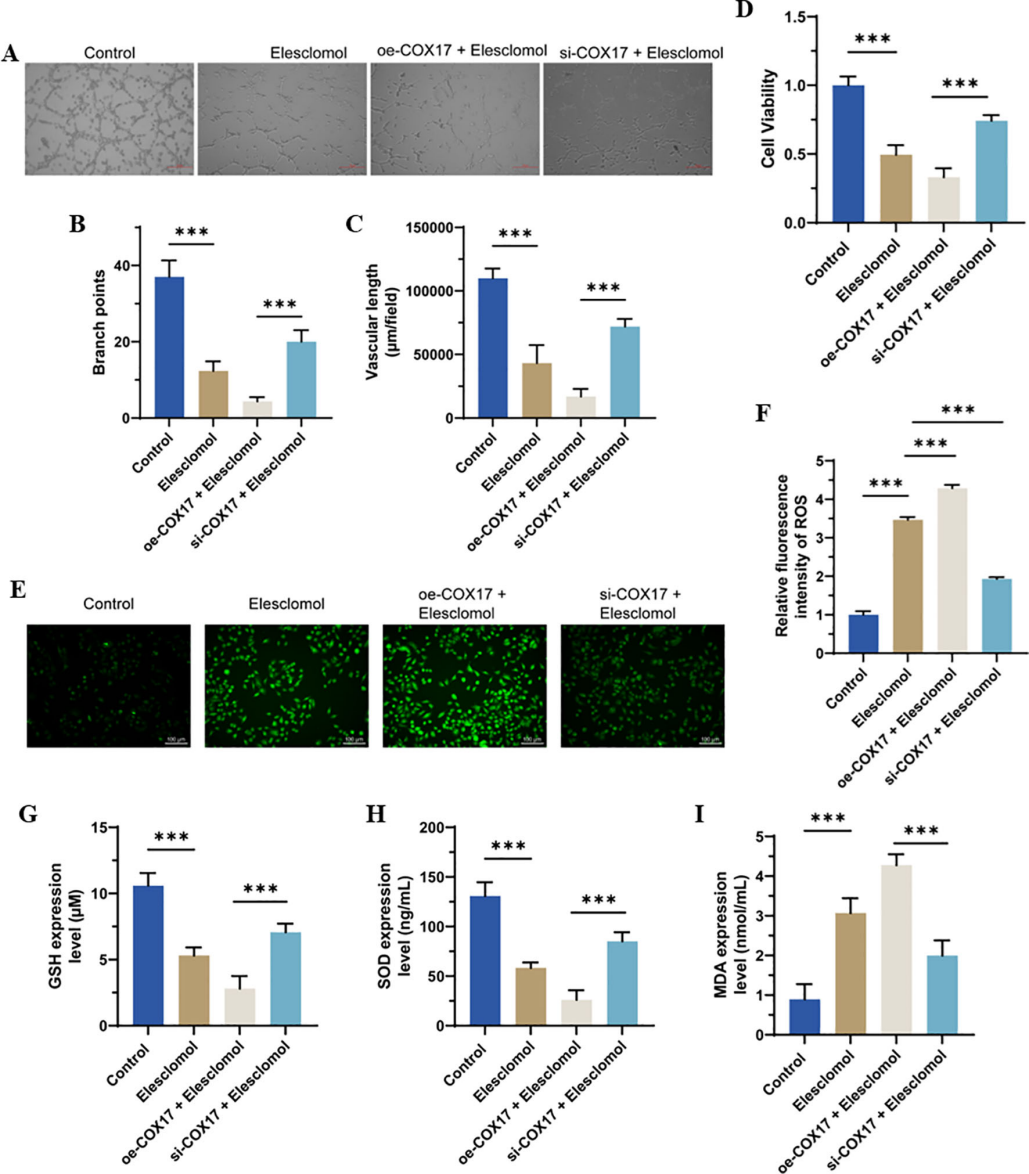
Conditioned media from COX17-modulated AGS cells affect angiogenesis-related phenotypes in HUVECs. **(A)** Representative tube formation images of HUVECs treated with conditioned media from each AGS group. **(B, C)** Quantification of branch points and total vascular length per field. **(D)** HUVEC viability determined by CCK-8 assay. **(E)** Representative DCFH-DA fluorescence images indicating intracellular ROS levels. **(F)** Quantitative analysis of ROS fluorescence intensity. **(G–I)** Levels of GSH, SOD, and MDA in HUVECs measured using commercial kits. Data are expressed as mean ± SD (*n* = 3). Statistical significance: ***P < 0.001.

To investigate whether COX17-associated changes in elesclomol-treated gastric cancer cells influence angiogenesis-related phenotypes, conditioned media from differently treated AGS cells were applied to HUVEC cultures. As shown in **Figures 4A–C**, HUVECs cultured with conditioned medium from elesclomol-treated AGS cells exhibited significantly impaired tube formation compared with the control conditioned medium group (*P* < 0.001). This inhibitory effect was further enhanced when HUVECs were exposed to conditioned medium from COX17-overexposed AGS treated with elesclomol (*P* < 0.001 vs. elesclomol alone). In contrast, conditioned medium from COX17-silenced AGS cells partially alleviated the suppression of angiogenic branching and tube length (both *P* < 0.001 vs. oe-COX17 + elesclomol). These results suggest that COX17-associated changes in elesclomol-treated AGS cells are linked to impaired angiogenesis-related phenotypes of HUVECs *in vitro*. Consistent with the morphologic data, the CCK-8 cell assay showed that conditioned medium from elesclomol-treated AGS cells reduced HUVEC viability compared with the control conditioned medium group (*P* < 0.001). This effect was further strengthened when COX17 was overexpressed in AGS cells (*P* < 0.001 vs. elesclomol). Conversely, COX17 knockdown partially restored HUVEC viability compared with the oe-COX17 + elesclomol CM group (*P* < 0.001) (**Figure 4D**). Thus, COX17 modulation in tumor cells may influence endothelial cell viability in the conditioned medium system.

ROS generation in HUVECs was then assessed using DCFH-DA fluorescence. As shown in **Figures 4E, F**, conditioned medium from elesclomol-treated AGS cells increased the intracellular ROS levels in HUVECs compared with the control conditioned medium group (*P* < 0.001). The highest ROS level was observed in the oe-COX17 + elesclomol group, whereas COX17 silencing markedly reduced ROS accumulation compared with the oe-COX17 + elesclomol group (*P* < 0.001). Quantitative biochemical assays further confirmed oxidative stress imbalance in HUVECs (**Figures 4G–I**). The antioxidant indicators GSH and SOD were significantly decreased following exposure to elesclomol (*P* < 0.001 vs. control) and reached their lowest levels in the oe-COX17 + elesclomol group (*P* < 0.001 vs. elesclomol alone). Conversely, MDA, a marker of lipid peroxidation, was significantly increased in the same group (*P* < 0.001). COX17 knockdown partially reversed these changes, resulting in higher GSH and SOD levels and lower MDA levels compared with the oe-COX17 + elesclomol group (all *P* < 0.001). Taken together, these results suggest that conditioned media from COX17-overexpressing AGS cells treated with elesclomol suppress endothelial cell proliferation and tube formation, at least in association with increased oxidative stress. These findings suggest that conditioned media from COX17-overexpressing, elesclomol-treated AGS cells inhibit the angiogenesis-related phenotypes of HUVECs *in vitro*.

### 3.5 *In vivo* validation of COX17 in elesclomol-induced tumor suppression in a gastric cancer xenograft model

**Figure 5:**
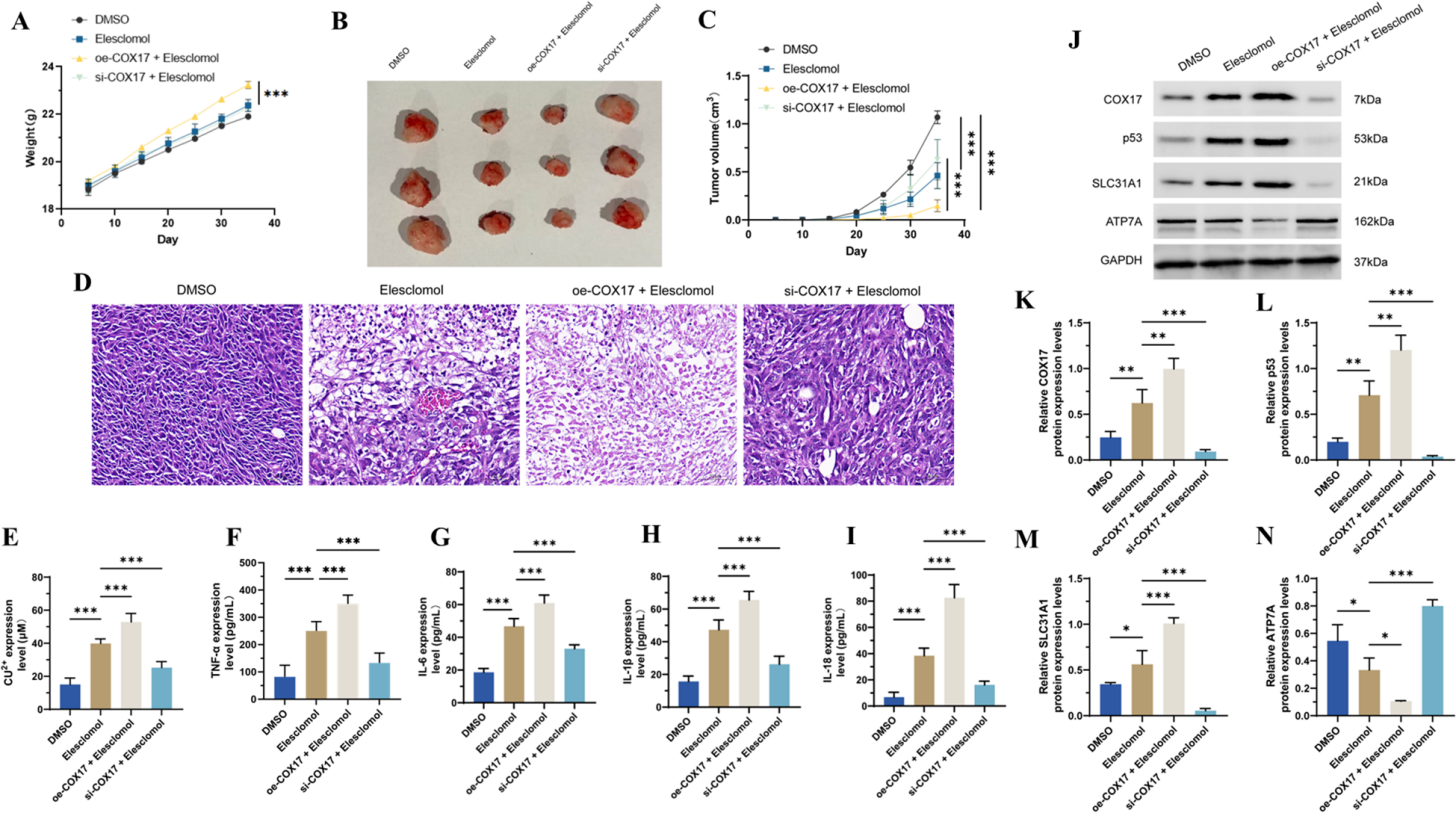
*In vivo* validation of COX17 in elesclomol-induced tumor suppression in a gastric cancer xenograft model. **(A)** Body weight curve of nude mice during 28 days of treatment. **(B)** Representative images of excised tumors from each group. **(C)** Tumor growth curve showing the volume changes over time. **(D)** H&E staining of xenograft tissues (scale bar = 50 μm). **(E)** Tumor copper ion concentration quantified by colorimetric assay. **(F–I)** Serum TNF-α, IL-6, IL-1β, and IL-18 levels determined by ELISA. **(J)** Representative Western blot of COX17, p53, SLC31A1, and ATP7A. **(K–N)** Quantitative densitometry of corresponding proteins normalized to GAPDH. Data are expressed as mean ± SD (*n* = 3); *P < 0.05, **P < 0.01, ***P < 0.001.

To evaluate the role of COX17 in elesclomol-induced tumor suppression *in vivo*, a subcutaneous xenograft model was established using AGS gastric cancer cells. Nude mice were randomly assigned to four groups—DMSO (control), elesclomol, COX17-overexpression + elesclomol, and COX17-knockdown + elesclomol—and treated for 28 days. As shown in **Figures 5A–C**, body weight gradually increased in all groups during the treatment period, although intergroup differences were observed. Mice in the DMSO group showed a relatively lower body weight, possibly reflecting greater tumor burden, whereas mice in the COX17 overexpression + elesclomol group maintained a relatively higher body weight during the study (*P* < 0.001), suggesting a better overall systemic condition. The elesclomol group showed moderate body weight changes, whereas COX17 knockdown slightly attenuated the body weight improvement observed after elesclomol treatment. Tumor growth was significantly inhibited in mice treated with elesclomol compared with the DMSO group. COX17 overexpression further enhanced the antitumor effect of elesclomol, producing the smallest tumor size and volume among all groups (*P* < 0.001 vs. elesclomol alone). In contrast, COX17 silencing attenuated the inhibitory effect of elesclomol on tumor growth (*P* < 0.001 vs. OE-COX17 + elesclomol), resulting in larger tumors than those in the COX17 overexpression + elesclomol group (**Figures 5B, C**).

Histopathological examination provided supportive tissue-level evidence. H&E staining showed more apparent structural disruption and necrotic changes in elesclomol-treated tumors than in control tumors. Tumors from the COX17 overexpression + elesclomol group exhibited more extensive degenerative and necrotic areas, whereas COX17 knockdown reduced the extent of tissue damage and preserved more viable tumor regions (**Figure 5D**). These histological findings are consistent with enhanced tumor injury after COX17 overexpression combined with elesclomol treatment. The colorimetric analysis of tumor tissues’ copper content revealed that elesclomol significantly increased copper accumulation compared with the DMSO group (*P* < 0.001). The highest copper concentration was observed in the COX17 overexpression + elesclomol group (*P* < 0.001 vs. elesclomol), whereas COX17 silencing markedly decreased the copper levels compared with the COX17 overexpression + elesclomol group (*P* < 0.001) (**Figure 5E**). Moreover, the ELISA assays of serum inflammatory cytokines showed that elesclomol treatment elevated the TNF-α, IL-6, IL-1β, and IL-18 levels (all *P* < 0.001 vs. DMSO), and these effects were further enhanced in the COX17 overexpression + elesclomol group. Conversely, COX17 knockdown attenuated elesclomol-induced cytokine release, shifting the cytokine levels toward those observed in the control group (*P* < 0.001) (**Figures 5F–I**).

Western blot analysis of xenograft tissues further confirmed the molecular changes observed *in vitro*. Elesclomol treatment increased the protein expression of COX17, p53, and SLC31A1 and decreased ATP7A expression compared with the control group (all *P* < 0.05). COX17 overexpression combined with elesclomol resulted in the greatest increase in COX17, p53, and SLC31A1 expression and the most pronounced reduction in ATP7A expression. In contrast, COX17 knockdown reduced the expression of COX17, p53, and SLC31A1 and partially restored ATP7A expression toward the control level (*P* < 0.05) (**Figures 5J–N**). Taken together, these *in vivo* findings suggest that COX17 enhances elesclomol-induced copper accumulation, tumor tissue injury, and tumor growth inhibition in AGS xenografts. These *in vivo* findings together suggest that COX17 enhances the antitumor effect of elesclomol in AGS xenografts, accompanied by increased copper accumulation, histological injury, inflammatory cytokine release, and changes in copper transport-related proteins. These results further support the involvement of the p53–COX17 axis in elesclomol-associated tumor suppression *in vivo*.

## 4 Discussion

Cuproptosis is a recently identified form of regulated cell death caused by copper accumulation and mitochondria protein lipoylation and is closely associated with metal homeostasis and mitochondria energy metabolism (18, 19). As a master regulator of stress responses and cellular metabolism, p53 plays pivotal roles not only in apoptosis and ferroptosis but also in maintaining mitochondrial function (20). COX17, a key copper chaperone that transfers Cu^+^ ions to cytochrome c oxidase in the mitochondrial intermembrane space, represents a potential node linking p53 signaling to copper metabolism (21). In this study, we found that p53 was associated with increased COX17 expression and may participate in the transcriptional regulation of COX17, thereby enhancing copper accumulation and increasing the sensitivity of gastric cancer cells to elesclomol-induced copper-associated cytotoxicity. Through a combination of *in vitro* and *in vivo* experiments, our data suggest that the p53–COX17 axis may serve as an important pathway integrating copper metabolism, oxidative stress, and tumor suppression.

Our initial results showed that COX17 alone had only a modest effect on basic cell viability, but it markedly influenced the response of gastric cancer cells to elesclomol treatment. This selective effect suggests that COX17 may not function simply as an oncogene or tumor suppressor per se, but rather as a copper-sensitizing modulator under copper-associated stress (22). Overexpression of COX17 markedly enhanced the cytotoxicity of elesclomol, whereas COX17 knockdown alleviated the drug’s effect. Mechanistically, COX17 is known to facilitate copper delivery to cytochrome c oxidase complexes and other mitochondrial cuproenzymes (23). Under conditions of elesclomol-induced copper stress, increased COX17 expression may promote mitochondrial copper handling and thereby aggravate copper-associated mitochondrial dysfunction. In canonical cuproptosis, excessive copper can bind to lipoylated TCA cycle proteins, such as DLAT and DLST, leading to protein aggregation, proteotoxic stress, Fe–S cluster protein loss, mitochondrial dysfunction, ROS accumulation, and ultimately cell death. Although these canonical events were not directly examined in the present study, our observations of increased intracellular copper accumulation, altered copper transport-related protein expression, reduced cell viability, and increased cell death are consistent with cuproptosis-associated copper-dependent cytotoxicity. These events were accompanied by increased SLC31A1 expression and reduced ATP7A expression, further supporting the notion that COX17 is associated with altered intracellular copper homeostasis. This mechanism is consistent with the model proposed by Tsvetkov et al. (24), which identified copper–lipoylated protein interactions as key events in cuproptosis, while our data further suggest that COX17 may act as an upstream modulator affecting cellular susceptibility to elesclomol-induced copper stress.

Further investigation revealed that p53 positively regulates COX17 expression. Overexpression of p53 increased the COX17 protein levels and suppressed the proliferation, migration, and invasion of AGS cells, whereas p53 silencing produced the opposite effects. ChIP-qPCR analysis showed the enrichment of p53 at the COX17 promoter region, suggesting that p53 may participate in the transcriptional regulation of COX17 expression. This finding is consistent with prior evidence that p53 can modulate mitochondrial respiration and oxidative phosphorylation by regulating genes involved in mitochondrial biogenesis and electron transport (25, 26). By upregulating COX17, p53 may enhance the mitochondrial copper-handling capacity of gastric cancer cells, thereby creating a metabolic vulnerability under copper stress (27). The ability of p53 to coordinate mitochondrial function and copper-related stress responses underscores its role as a metabolic gatekeeper linking stress signaling to metal-dependent cell death pathways (25, 28, 29).

Mechanistic epistasis experiments further supported the functional relationship between p53 and COX17. The cytotoxic effect associated with p53 overexpression under elesclomol treatment was substantially attenuated when COX17 was silenced, whereas reintroduction of COX17 in p53-deficient cells partially restored elesclomol sensitivity. This genetic interdependence suggests that COX17 is an important downstream mediator of p53-mediated copper stress responses. It may also help explain why p53 status influences copper-associated cytotoxicity in tumors: cells with functional p53 may exhibit a higher COX17 expression and enhanced sensitivity to copper ionophores, whereas p53-deficient cells may show reduced copper trafficking capacity and relative resistance. This observation is consistent with previous studies showing that p53 can regulate mitochondrial oxidative stress, although the relationship between p53 and copper metabolism may vary depending on copper load, mitochondrial metabolic state, and tumor type (30, 31). Such context-dependent differences may be particularly relevant in gastric cancer cells, which often rely on mitochondrial metabolic pathways and may therefore be vulnerable to p53–COX17-associated copper stress. Although COX17 modulation was associated with changes in p53 protein expression in our study, the current data do not support the direct transcriptional regulation of p53 by COX17. Because COX17 is a mitochondrial copper chaperone rather than a DNA-binding transcription factor, the observed increase in p53 expression following COX17 overexpression is more likely related to enhanced copper-associated mitochondrial stress, ROS accumulation, or secondary stress response signaling. Therefore, the relationship between p53 and COX17 may involve a feed-forward or stress-associated feedback loop, in which p53 transcriptionally promotes COX17 expression, whereas COX17-mediated copper dysregulation secondarily influences p53 activation. Additional promoter–reporter assays, COX17 mRNA analyses, and time-course experiments will be required to further clarify the directionality and mechanistic basis of this interaction.

Our conditioned medium and endothelial experiments suggested that COX17- associated changes in elesclomol-treated gastric cancer cells may also affect angiogenesis-related phenotypes. Conditioned media from elesclomol-treated, COX17-overexpressing AGS cells impaired HUVEC proliferation and tube formation, accompanied by increased intracellular ROS, elevated MDA levels, and reduced antioxidant molecules GSH and SOD. These results suggest that factors released from elesclomol-treated tumor cells may propagate redox imbalance in endothelial cells and impair angiogenesis-related behavior. However, because conditioned medium experiments cannot fully distinguish between tumor cell-derived soluble mediators and potential residual drug-related effects, these findings should be interpreted as angiogenesis-related phenotypic changes rather than direct mechanistic proof of anti-angiogenesis. This observation is in line with studies in other regulated cell death models, including ferroptosis, in which lipid peroxidation and ROS-mediated stress can influence vascular remodeling (32–34). Our *in vivo* data also showed more extensive necrotic changes in COX17-overexpressing tumors treated with elesclomol, supporting the possibility that COX17-associated copper stress may influence both tumor cell survival and the tumor microenvironment (35–37). Further studies examining angiogenesis-related factors and vascular markers will be required to clarify this mechanism.

The xenograft experiments provided *in vivo* support for the role of COX17 in elesclomol-associated tumor suppression. Mice bearing COX17-overexpressing tumors and treated with elesclomol showed the most pronounced tumor growth inhibition and relatively better maintenance compared with the other groups. This suggests that enhanced tumor suppression may reduce tumor-burden-related systemic deterioration in this model. Tumors with higher copper accumulation also showed elevated TNF-α, IL-6, IL-1β, and IL-18 levels, indicating that elesclomol-associated copper stress may be accompanied by inflammatory responses *in vivo*. Molecular analyses revealed a consistent protein expression pattern, including the upregulation of p53, COX17, and SLC31A1, together with suppression of ATP7A, mirroring the *in vitro* observations. These findings together support a model in which p53-associated COX17 upregulation promotes copper accumulation and enhances elesclomol-associated antitumor effects in gastric cancer xenografts (29). Mechanistically, the observed outcomes may involve several interconnected processes. First, p53 may upregulate COX17 to promote copper handling and sensitize cells to elesclomol-induced copper stress (27, 38). Second, excessive copper may impair mitochondrial function and promote ROS accumulation, contributing to cell-death-associated changes (36, 39). Third, oxidative-stress-related factors from treated tumor cells may influence endothelial cell behavior and contribute to impaired angiogenesis-related phenotypes (40). The net effect is enhanced tumor growth suppression, in which p53 acts as an upstream regulatory factor, COX17 as a copper-metabolism-related mediator, and elesclomol as a pharmacological amplifier.

Despite these findings, several limitations should be acknowledged. First, although our data support the involvement of COX17-mediated copper handling in elesclomol-induced cytotoxicity, canonical cuproptosis markers, including FDX1, DLAT/DLST, global protein lipoylation, lipoylated protein aggregation, and Fe–S cluster protein changes, were not directly examined in the present study. Future studies should include these markers to more specifically define cuproptosis. Second, TUNEL staining detects DNA fragmentation/cell death and is not specific for cuproptosis. Therefore, the TUNEL results in this study should be interpreted as evidence of cell-death-associated DNA fragmentation rather than as a direct marker of cuproptotic cell death. Third, copper chelator rescue experiments using agents such as tetrathiomolybdate or bathocuproine disulfonate were not performed, and such experiments will be important to confirm copper dependence. Fourth, this study was mainly based on the AGS cell line, which has a wild-type p53 background. Because p53 mutation is common in gastric cancer, validation in additional gastric cancer cell lines, especially p53-mutant models such as MKN45 or SNU-1, is needed to determine the generalizability of the p53–COX17 axis. Fifth, although ChIP-qPCR suggested p53 enrichment at the COX17 promoter region, additional assays, including COX17 mRNA detection and promoter luciferase reporter assays, are needed to further verify transcriptional regulation. Sixth, the anti-angiogenic effect was inferred from conditioned medium-based HUVEC assays and oxidative stress markers. Future studies should include residual elesclomol controls, angiogenesis-related mediators such as VEGF, ANGPT2, and HIF-1α, and tissue vascular markers such as CD31 to better define this mechanism. Finally, clinical validation was not included in the present study. Further analysis of COX17 expression in gastric cancer and adjacent normal tissues, as well as IHC validation of p53, COX17, SLC31A1, and ATP7A in xenograft or clinical tumor tissues, will be required before proposing COX17 as a biomarker or patient stratification indicator.

## 5 Conclusion

In summary, this study suggests a potential regulatory link between p53, COX17, and copper metabolism in gastric cancer. p53 may positively regulate COX17 expression, thereby enhancing copper accumulation and increasing the sensitivity of AGS gastric cancer cells to elesclomol-induced copper-associated cytotoxicity. COX17 upregulation was associated with increased oxidative stress, reduced tumor cell viability, enhanced DNA fragmentation/cell death, impaired angiogenesis-related phenotypes *in vitro*, and stronger tumor growth inhibition *in vivo*. These findings support the involvement of the p53–COX17 axis in elesclomol-associated antitumor effects in gastric cancer. However, because additional cuproptosis-specific markers, copper chelator rescue experiments, p53-mutant gastric cancer models, and clinical validation were not included in the present study, the translational significance of this pathway requires further investigation. Future studies should clarify the precise molecular events downstream of COX17-mediated copper handling, validate this mechanism in gastric cancer models with different p53 backgrounds, and evaluate whether COX17 expression may help predict sensitivity to copper-ionophore-based therapeutic strategies.

## Data Availability

The original contributions presented in the study are included in the article/supplementary material. Further inquiries can be directed to the corresponding author.
